# Relative influence of wild prey and livestock abundance on carnivore‐caused livestock predation

**DOI:** 10.1002/ece3.6815

**Published:** 2020-09-24

**Authors:** Gopal Khanal, Charudutt Mishra, Kulbhushansingh Ramesh Suryawanshi

**Affiliations:** ^1^ Post‐Graduate Program in Wildlife Biology and Conservation National Centre for Biological Sciences Bangalore India; ^2^ Center for Wildlife Studies Wildlife Conservation Society‐India Program Bangalore India; ^3^ Department of National Parks and Wildlife Conservation Ministry of Forests and Environment Government of Nepal Kathmandu Nepal; ^4^ Centre for Ecological Studies Lalitpur Nepal; ^5^ Nature Conservation Foundation Mysore India; ^6^ Snow Leopard Trust Seattle WA USA

**Keywords:** conservation conflict, human carnivore conflict, large mammalian carnivore, livestock depredation, Nepal, Shey Phoksundo National Park, snow leopard

## Abstract

Conservation conflict over livestock depredation is one of the key drivers of large mammalian carnivore declines worldwide. Mitigating this conflict requires strategies informed by reliable knowledge of factors influencing livestock depredation. Wild prey and livestock abundance are critical factors influencing the extent of livestock depredation. We compared whether the extent of livestock predation by snow leopards *Panthera uncia* differed in relation to densities of wild prey, livestock, and snow leopards at two sites in Shey Phoksundo National Park, Nepal. We used camera trap‐based spatially explicit capture–recapture models to estimate snow leopard density; double‐observer surveys to estimate the density of their main prey species, the blue sheep *Pseudois nayaur*; and interview‐based household surveys to estimate livestock population and number of livestock killed by snow leopards. The proportion of livestock lost per household was seven times higher in Upper Dolpa, the site which had higher snow leopard density (2.51 snow leopards per 100 km^2^) and higher livestock density (17.21 livestock per km^2^) compared to Lower Dolpa (1.21 snow leopards per 100 km^2^; 4.5 livestock per km^2^). The wild prey density was similar across the two sites (1.81 and 1.57 animals per km^2^ in Upper and Lower Dolpa, respectively). Our results suggest that livestock depredation level may largely be determined by the abundances of the snow leopards and livestock and predation levels on livestock can vary even at similar levels of wild prey density. In large parts of the snow leopard range, livestock production is indispensable to local livelihoods and livestock population is expected to increase to meet the demand of cashmere. Hence, we recommend that any efforts to increase livestock populations or conservation initiatives aimed at recovering or increasing snow leopard population be accompanied by better herding practices (e.g., predator‐proof corrals) to protect livestock from snow leopard.

## Graphical abstract

Conservation conflict over livestock depredation is one of the key drivers of large mammalian carnivore declines worldwide. We compared whether the extent of livestock predation by snow leopard differs at two sites in relation to the density of wild prey blue sheep, livestock, and snow leopard. The number of livestock lost per household to snow leopards was seven times higher in Upper Dolpa, the site which had higher snow leopard density (2.51 snow leopards per 100 km^2^), higher livestock density (17.21 livestock per km^2^) than Lower Dolpa (1.21 snow leopards per 100 km^2^; 4.5 livestock per km^2^). Our results suggest that livestock predation levels may largely be determined by the abundance of the predator and the livestock.

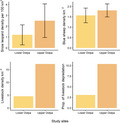

## 1 INTRODUCTION

Large mammalian carnivores are among the most threatened group of species with over 60% of them facing high risk of extinction (Ripple et al., 2014). While habitat loss, fragmentation, poaching, and prey depletion continue to cause their populations to decline (Cardillo et al., 2004; Chapron et al., 2008; Wolf & Ripple, 2016), retaliatory killing over livestock predation is perhaps the most widespread and direct threat to their conservation (Inskip et al., 2014). Livestock depredation compromises the livelihoods of often marginalized communities and erodes human tolerance of carnivores (Inskip et al., 2016; Mishra, 1997; Thirgood et al., 2005; Treves & Karanth, 2003). It is therefore critical to mitigate the conflicts surrounding livestock depredation for ensuring sustainable livestock production by pastoral communities and continued survival of carnivore populations, especially for wide‐ranging species that occur outside protected areas. Reducing livestock depredation by carnivores requires an understanding of factors affecting their predation behavior.

Multiple factors influence the extent of carnivore‐caused livestock predation, including livestock husbandry practices (Kuiper et al., 2015; Ogada et al., 2003; Woodroffe et al., 2007), wild prey availability (Meriggi et al., 1996; Odden et al., 2008), habitat type and structure (Miller et al., 2015), behavioral characteristics of the predator (Lucherini et al., 2018), and predator abundance (Lesilau et al., 2018; Miller et al., 2015; Weise et al., 2018). Understanding the interplay between predator, wild prey, and livestock density is important for identifying mitigation measures.

Density of wild herbivore prey is known to be a critical determinant of carnivore density (Carbone & Gittleman, 2002; Karanth et al., 2004; Suryawanshi et al., 2017). However, the role of wild herbivore density in determining the extent of livestock predation by carnivores is debatable (Bagchi & Mishra, 2006; Khorozyan et al., 2015; Meriggi et al., 1996; Meriggi & Lovari, 1996; Soofi et al., 2019; Suryawanshi et al., 2017). Studies investigating the impact of livestock abundance on predation levels have shown higher intensities of depredation in areas of higher livestock densities (Pimenta et al., 2018). Despite a range of field studies and reviews examining patterns of livestock depredation by large carnivores (van Eeden et al., 2018; Inskip & Zimmermann, 2009; Janeiro‐Otero et al., 2020; Weise et al., 2018), our knowledge of the relative impact of wild prey and livestock abundance on livestock predation is still limited.

The snow leopard *Panthera uncia* is listed as Vulnerable in the IUCN Red list of Threatened Species and occurs in 12 countries across the Himalaya and high mountains of Central Asia (McCarthy et al., 2017). Fewer than 4,000 adult snow leopards are believed to occur in the wild and little is known about their population trends (Snow Leopard Working Secretariat, 2013; Suryawanshi et al., 2019). Pastoralism is the dominant form of land use and economy across the snow leopard distribution range in Central and South Asia (Mishra et al., 2009). As its distribution range overlaps extensively with the pastoral production landscapes, livestock predation by snow leopard is ubiquitous and is of high concern for pastoral communities. Among other factors such as habitat loss and decline of prey, livestock depredation has been the key factor driving its endangerment through retaliatory killings while also imposing significant economic costs on marginalized herder communities (Aryal et al., 2014; Hussain, 2003; Ikeda, 2004; Johansson et al., 2015; Li et al., 2013; Mishra, 1997). Herders have been found to incur high losses, up to 12% of their livestock holdings annually, to snow leopard and sympatric predators, which sometimes amounts up to 50% of the average annual household income (Mishra, 1997; Oli et al., 1994). Livestock loss causes serious hostility among herder communities, often resulting in persecution of the snow leopard (Oli et al., 1994).

A few studies that have tried to identify the causes of livestock predation by snow leopards have found a range of factors influencing the extent of livestock predation such as wild prey density, livestock density, herding practices, and habitat type (Alexander, et al., 2015; Alexander, et al., 2015; Bagchi & Mishra, 2006; Bagchi et al., 2020; Chetri et al., 2017, 2019; Jackson et al., 1996; Rashid et al., 2020; Suryawanshi et al., 2013, 2017). These studies have generated an understanding of spatial and temporal patterns of livestock predation, providing insights into the location and season for prioritizing mitigation measures. Suryawanshi et al. (2017) showed that the extent of predation on livestock could increase with livestock density as well as wild prey density via increased snow leopard density. However, relative influence of wild prey and livestock density on livestock predation by snow leopards is still unclear as livestock predation can often be context‐dependent with site specific idiosyncrasies in habitat characteristics and management interventions (Chetri et al., 2019).

The Himalayan and trans‐Himalayan habitats of Shey Phoksundo National Park, Nepal, which vary in habitat characteristics and livestock management practices offer an interesting opportunity to examine such effects. Here, we report on the extent of snow leopard predation on livestock in relation to wild prey and livestock density in two sites, Upper Dolpa and Lower Dolpa, that are broadly characterized by the trans‐Himalayan and Himalayan habitats, respectively. We also examined the factors influencing household level variation in livestock depredation by snow leopard.

## 2 MATERIALS AND METHODS

### 2.1 Study area

**Figure 1:**
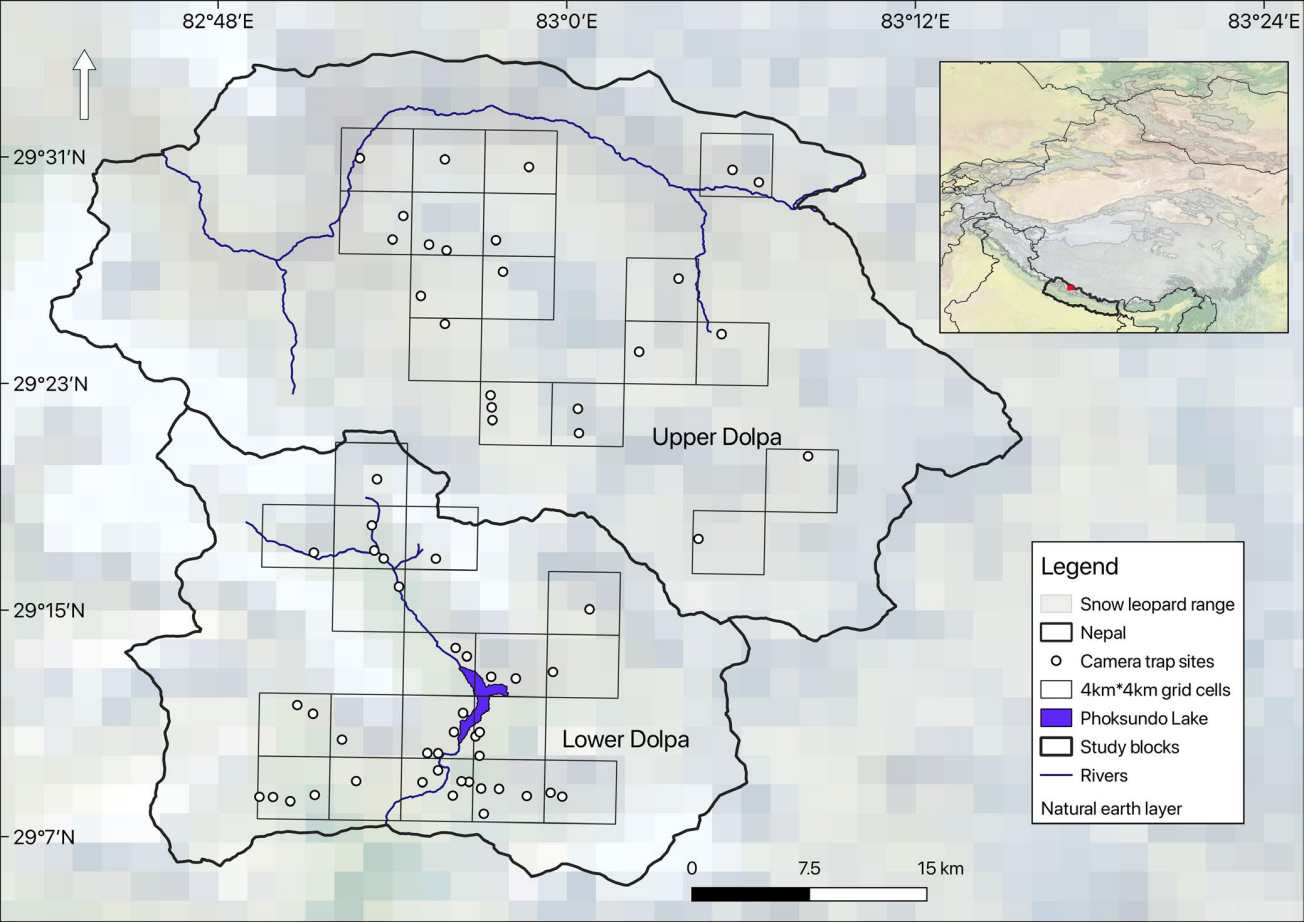
Study sites including the Himalayan Lower Dolpa and trans‐Himalayan Upper Dolpa within Shey Phoksundo National Park, Nepal, where snow leopard *Panthera uncia* and prey abundance estimation were undertaken

This study was conducted at two sites, the Lower Dolpa and the Upper Dolpa in Shey Phoksundo National Park, Nepal (29°15′–29°45′ N and 83°08′–83°31′ E; Figure 1). Located in western part of Nepal, the park covers an area of 3,555 km^2^ with elevation ranging from 2,130 m in Ankhe to 6,883 m at the summit of Kanjirowa Mountain. The park contains the transition from a monsoon dominated climate with 1,500 mm of annual precipitation in the south (Suligad) to an arid climate with less than 500 mm a year in the northern slopes.

The main mammalian fauna includes snow leopard (Panthera uncia), red fox (Vulpes vulpes), jackal (Canis aureus), gray wolf (Canis lupus), Himalayan marmot (Marmota himalayana), musk deer (Moschus chrysogaster) and Himalayan goral (Naemorhedus goral), and bharal (blue sheep Pseudois *nayaur*). The boundary of the two sites is separated by a ridgeline and mountain pass of about 5,000 m from mean sea level. Lower Dolpa (c. 600 km^2^) lies in the southern part of the park and is characterized by a Himalayan ecosystem with tree line vegetation, and mixed broadleaved conifer species dominating up to 3,600 m elevation. Upper Dolpa (c. 800 km^2^) lies in the north and is a rain shadow zone bordering China. Unlike Lower Dolpa, Upper Dolpa is above tree line, characterized by the trans‐Himalayan ecosystem with less than 500 mm annual rainfall and dominated by dry alpine steppe, meadows rich in sedges and graminoids such as *Stipa*, *Carex,* and *Kobresia*, and shrubs such as *Caragana brevifolia* and *Lonicera spinosa* on dry sites. Human population in Lower Dolpa is about 500 in four villages, whereas Upper Dolpa has a population of 750 in 18 scattered villages (Table 1). In Lower Dolpa, people have largely stopped rearing small livestock including sheep *Ovis aries* and goats *Capra hircus* (except 4–5 households) due to increasing livelihood opportunities in tourism, but continue to rear large livestock such as yaks *Bos grunniens*, cattle‐yak hybrids (dzo, jhopas, *Bos* spp.), and horses *Equus ferus caballus*. In Upper Dolpa, small livestock species dominate the holding size but large livestock species are also reared in high number. In winter, people of Rigmo village descend to low elevation areas (below 3000 m) with all of their livestock to avoid heavy snowfall. However, people from Upper Dolpa generally do not migrate to low elevation areas (but some families who have large number of large livestock, mostly yaks, migrate with their stock). Upper Dolpa in addition to having higher livestock density also has higher holding size per household, more scattered villages, more sheep and goats, and higher number of grazing days in pasture far from home compared to Lower Dolpa (Table 1).

**Table 1:** A comparison of the two study sites where the abundances of snow leopards, abundance, wild prey, and livestock were assessed, and the extent of livestock predation was compared. The study sites include Upper Dolpa and Lower Dolpa in Shey Phoksundo National Park, Nepal

Description	Lower Dolpa	Upper Dolpa
Location	29.19° N, 82.92° E	29.42° N, 82.99° E
Altitude (m)	3,060–6,883	3,145–6,244
Number of human settlements	5	18
Total human population	550	2,549
Total households	108	664
Total livestock population	1,607	12,911
Cattle	50	747
Yak/horse	1,181	1,740
Goat	91	3,362
Sheep	285	7,062
Average livestock holding size	18.71	40.20

### 2.2 Camera trapping survey of snow leopard

We deployed 65 camera traps (Model HC550; Reonyx Inc, and Cuddeback IR) at 62 stations in the two study sites over an area of approximately 700 km^2^ (Figure 1) from November 2017 through April 2018. Details of site‐wise sampling duration, average trap spacing, and camera array areas are provided in Table 2. To ensure systematic placement of camera traps, we overlaid the study area with 4 × 4 km^2^ square grid cells for camera trap deployment. This grid cell size was chosen to be small enough to avoid holes for snow leopards to go undetected and large enough to have multiple spatial recaptures of individuals. Before deploying camera traps, areas with higher probability of encountering snow leopard signs such as human trails, well‐defined and narrow ridgelines, valley bottoms, scent marking sites or immediately adjacent to frequently scent‐sprayed rocks and scrapes were identified through preliminary sign surveys (Jackson et al., 2006). Camera traps were then set up in such locations within grid cells maintaining intertrap distance of 1–3 km in order to maximize recaptures at different camera trap stations considering recommendations of the simulation studies (Sollmann et al., 2012).

**Table 2:** Summary of site‐wise survey duration, number of camera stations, and camera array polygon, and estimated parameters of interest: density (D) per 100 km2, its lower confidence interval (LCL)‐upper confidence level (UCL), capture probability (g0), spatial scale movement parameter (σ)

Description	Lower Dolpa	Upper Dolpa
Survey duration	2 Nov. 2017–14 Apr. 2018	8 Nov. 2017–25 Apr. 2018
Camera trap stations	39	23
Camera trap polygon Area	350 km^2^	350 km^2^
Camera trap spacing	1,608.95 m	2,913. 24 m
Density (SE)	1.21 (0.47)	2.51 (0.79)
LCL‐ UCL	0.58–2.54	1.36–4.60
g0 (SE)	0.03 (0.007)	0.04 (0.01)
σ (SE)	4,846 (499)	2,172 (291)

### 2.3 Wild prey (blue sheep) survey

We used the “double‐observer” survey method to estimate the abundance of blue sheep (Forsyth & Hickling, 1997; Suryawanshi et al., 2012). This method is analogous to two sample capture–mark–recapture (CMR) technique of animal abundance estimation (Williams et al., 2002). The logic is that individual animals are difficult to uniquely identify and mark in mountain ungulate species but groups or herds can be identified uniquely based on group characteristics, sighting location and time etc. Hence, traditional CMR can be still used at the group level, the unit of analysis being the group or herd. In this method, the study area is divided into smaller blocks where surveys (combination of trails and observation points) can be done ensuring complete visual coverage. Two observers or two teams of observers independently search and count the herds and numbers of animals along specific trails or routes separated by about 15–20 min. Both observer teams record sufficient information on each of the ungulate sighting (e.g., herd size, geographic location, time of the sighting, distance to the herd location, age‐sex composition of the herd) to allow them to later identify the common (recaptured herds) and unique herds. The key assumptions of this form of double‐observer survey are that complete visual coverage of survey block is possible, common groups are not misidentified, and there is no group fission or fusion during the duration of the two surveys (Suryawanshi et al., 2012).

To conduct the field surveys, we first mapped 5–7 blocks of 30–50 km^2^ in both Upper and Lower Dolpa. These blocks were delimited using geographic features such as rivers, ridgelines, and watershed boundary. Human‐used trails, valley bottoms, and ridgelines were mapped as potential survey routes within these sub‐blocks. Two observers equipped with either binoculars or spotting scope then conducted double‐observer surveys along these routes spacing themselves by 15–20 min. Information on herd size, age‐sex composition of the herd, sighting time and location and any particular characteristics and composition of the herd (e.g., male only groups) were used for verifying unique and common (recapture) herds and to avoid double counting. Surveys were conducted between 19 February 2018 and 26 April 2018 when blue sheep movement between adjacent blocks was generally low due to high accumulation of snow.

### 2.4 Livestock depredation surveys

We conducted household level interviews using semi‐structured questionnaire forms to gather information on species wise livestock holdings, grazing practices and livestock lost to snow leopards. A snowball sampling approach was used to sample households, where a respondent was asked to introduce another respondent (Goodman, 1961). A semi‐structured questionnaire form included questions pertaining to socio‐demographic characteristics of the respondents, livestock loss incurred due to snow leopards or other wild predators, monetary loss experienced and perception toward snow leopard conservation (Table S3). We asked the respondents to report the livestock losses incurred during the calendar year 2017. For each reported case of livestock depredation, additional information on the type of livestock lost, the month, date, time, and location was recorded when available. Out of 108 households in Lower Dolpa, 52 households were sampled and in Upper Dolpa 118 households were sampled out of 664 households (Central Bureau of Statistics, 2012). A pilot survey of twenty households was conducted to ensure that the questions were simple enough for respondents to understand clearly. Total livestock population size was obtained from the report of Department of Livestock Services, under the Ministry of Agriculture, Land Management and Cooperatives, Government of Nepal (Government of Nepal, 2017).

### 2.5 Data analysis

#### 2.5.1 Snow leopard population density

We used the maximum likelihood based spatially explicit capture–recapture (SECR) approach to analyze the spatial capture recapture data of snow leopards obtained from camera traps for density estimation. SECR is a spatially explicit hierarchical modeling process, which combines a state model and an observation model. The state model defines the distribution of animal activity centers in the landscape, and the observation model or spatial detection model describes the probability of capturing an animal at a certain trap to the distance of the trap from a mid‐point in individuals activity center or home range. Species density is then defined as the intensity of activity centers or spatial point pattern (home range centers). The joint fitting of the state and observation models provides an estimate of population density (Borchers & Efford, 2008; Royle et al., 2014). All analyses were conducted with the software package *secr* in R (Efford, 2018). The data input included the spatial capture recapture history of each identified snow leopard individual and trap layout information (geographic locations of each trap stations). Individual snow leopard IDs were prepared based on their pelage, forehead, and tail pattern (Alexander, et al., 2015; Alexander, et al., 2015; Jackson et al., 2006). At least two observers carefully reviewed photographic records of snow leopard captures, aided by video records if available. Any discrepancy in identification was jointly reviewed by the observers and consensus was reached on the individual IDs. To assess their accuracy level in being able to correctly identify individual snow leopards from capture images, both observers independently conducted self‐evaluations using the Snow Leopard Identification and Training Toolkit developed by the Global Snow Leopard Ecosystem Programme (GSLEP) http://camtraining.globalsnowleopard.org/ (Johansson et al., 2020). This tool kit uses a camera trap database of known individuals from zoos. The accuracy level for both observers was 93.88% and 92.78%, respectively, for 100 photographs each. We assumed that this level of accuracy was adequate for arriving at abundance estimates that were not significantly biased due to identification errors.

Each unique individual snow leopard from the field dataset was checked for recapture at the same station and at the other stations and across sites. Each day (24‐hr period) was considered as a unique sampling occasion (Karanth & Nichols, 1998). Spatial capture–recapture history of each individual snow leopard was prepared for the first 90 sampling occasions and included for analysis in order to try and meet the closure assumption.

For each study site, data were analyzed separately to estimate the parameters of interest: density (D), capture probability of an individual snow leopard at its activity center (g0), and the spatial scale over which detection probability declines as the distance between an individual's activity center to the camera trap station increases (σ). Variability in sampling effort may negatively bias density estimates and reduce the ability to explain variation in detection probability, so we accounted for variable sampling effort by using the number of days each sampling detector was active (Efford, 2018). We compared the fit of three detection functions: half normal, hazard rate, and exponential for the observation model to identify an appropriate function for capture probability using Akaike's information criterion, corrected for small sample sizes (AIC_c_). For both study sites, half‐normal detection function showed better fit. For all subsequent analysis, a half‐normal detection function was used for the observation model and a homogeneous Poisson distribution was used for the state model, which assumes latent activity centers are distributed evenly across the state space (Borchers & Efford, 2008). For both study sites, we fitted five plausible models for different detection functions and estimated the parameter based on the best model, assessed their relative support using Akaike's information criterion, corrected for small sample sizes (AICc) (Burnham & Anderson, 2002) and used the best supported model to make inference on density estimates (Tables S1a & S1b). We did not use any site covariates to model spatial variation in density since our primary goal was to obtain robust estimate of density for the two sites rather than spatial variation within them.

#### 2.5.2 Wild prey abundance and density

The total number of blue sheep herds was estimated using the Chapman's bias‐corrected estimator (Chapman, 1951). The total abundance of blue sheep was estimated as product of estimated number of herd and the mean herd size. Density of blue sheep within each site was calculated by dividing the double‐observer estimate of blue sheep abundance by the estimated area covered by the double‐observer surveys following Suryawanshi et al. (2012). The area of individual sub‐block was summed to get the total sampled area for both Upper and Lower Dolpa site separately. Freely available Google earth images and Quantum Geographic Information System software (QGIS) were used to map the sampled area.

#### 2.5.3 Livestock density and livestock predation

Livestock density was calculated by dividing the total livestock population by the total livestock grazing area, which was mapped in field with the help of local herders. Two metrics of livestock predation were calculated, (a) proportion of livestock holding lost per household, which was calculated as the total reported livestock lost by a household over a year (2017) divided by total livestock owned by the household, and (b) proportion of livestock holding lost at site level, which was calculated as the sum total of all livestock lost by all sampled households at a site divided by the total number of livestock owned by all sampled households at that site.

#### 2.5.4 Relationship between livestock predation, snow leopard density, wild prey density, livestock density, and household characteristics

We plotted simple bar graphs with standard deviations to assess whether densities of snow leopard, wild prey, and livestock differed between two sites. We also assessed whether the sites differed in total proportion of livestock lost and per household livestock lost. Snow leopard occupancy status remained fairly constant over two time periods November 2017–January 2018, when camera trap data surveys were conducted and February 2018–April 2018, when wild prey population sampling was done (Table S2). We have therefore assumed that no biases resulted due to the difference in time periods between when the camera trapping was done for snow leopard density estimation and the double‐observer surveys were conducted for wild prey density estimation. We ran generalized linear models (GLMs) with a Poisson error distribution and log link function to test the influence of three household level factors‐total livestock holding, number of small bodied livestock (goat and sheep) in total livestock holding, number of large bodied livestock to total holding and grazing days in pasture on household level variation in livestock depredation.

## 3 RESULTS

### 3.1 Snow leopard density

Overall, a total of 50 detections of 14 unique snow leopard adults were obtained from over 90 occasions at 23 camera traps stations in Upper Dolpa. In Lower Dolpa, from 39 traps stations, 48 detections of 7 unique snow leopard adults were obtained over 90 occasions. Based on the closure test function available in “secr” package, the sampled population appeared to be closed for both Upper Dolpa (*Z* = −0.97, *p* = .16) and Lower Dolpa (*Z* = −0.36, *p* = .35).

For both sites, the best model based on AICc included a constant capture probability (g0) and movement parameter (σ) with half‐normal detection as a detection function (Table S1). Estimated snow leopard density was 1.21 individuals per 100 km^2^ (95% CI 0.58–2.54) for Lower Dolpa and 2.51 individuals per 100 km^2^ (95% CI 1.36–4.60) for Upper Dolpa. The detection probability at activity center (g0) was 0.004 (Upper Dolpa), and 0.003 (Lower Dolpa) and the function of movement (σ) was estimated at 2,172 m (Upper Dolpa) and 4,846 m (Lower Dolpa; Table 2).

### 3.2 Wild prey blue sheep density

The estimated wild prey density was 1.81 blue sheep per square kilometer (95% CI = 1.49–2.11) for Upper Dolpa, whereas it was 1.57 blue sheep per square kilometer (95% CI = 1.22–1.91) in Lower Dolpa (Table 3). The mean group size of blue sheep herds was similar for both sites (16 individuals in Upper Dolpa and 16.47 individuals in Lower Dolpa). The estimated number of blue sheep groups was 34 for Lower Dolpa and 36 for Upper Dolpa. For Lower Dolpa, the detection probability was 0.67 and 0.70 for first observer and second observer, respectively, and it was 0.78 and 0.75 for first observer and second observer for Upper Dolpa.

**Table 3:** Results of double‐observer surveys of blue sheep in Upper and Lower Dolpa of Shey Phoksundo National Park, Nepal, between November 2017 and April 2018

Variables	Lower Dolpa	Upper Dolpa
No. of groups sighted by both observers	16	21
No. of groups sighted by first observer only	7	7
No. of groups sighted by second observer only	8	6
Estimated population	549	591
Variance in estimated population	3,585.29	2,642.61
Estimated detection probability Obs 1	0.667	0.778
Estimated detection probability Obs 2	0.696	0.750
Total area (km^2^)	350	328
Density	1.57 (95% CI = 1.22–1.91	1.81 (95% CI = 1.49–2.11)

### 3.3 Livestock population, density, and depredation

The total livestock population of Upper Dolpa was approximately 13,000 and Lower Dolpa was approximately 1,600 (Table 1). Livestock density was almost fourfold higher in Upper Dolpa (17.21 per square kilometer) as compared to Lower Dolpa (4.51 animals per km^2^) (Table 4). A total of 487 livestock were reportedly lost to snow leopard in Upper Dolpa and 30 livestock in Lower Dolpa in the year of 2017. Small stock (goats and sheep) accounted 90 percent of the total loss (Table 4). The proportion of total livestock holding lost to snow leopard was higher in Upper Dolpa (10.38% of livestock holding) in comparison to Lower Dolpa (3.08% of total livestock holding).

**Table 4:** Total livestock population, density, and reported livestock depredation by snow leopard in Upper and Lower Dolpa sites for a calendar year 2017

Description	Lower Dolpa	Upper Dolpa
Area (km^2^)	350	750
Total livestock population	1,607	12,911
Average holding size	18.71	40.20
Livestock density	4.59	17.21
Small bodied stock (goat and sheep) loss	2	463
Large bodied stock loss	28	24
Total livestock loss for a calendar year 2017	30	487
Proportion of livestock loss at site (% of total livestock holding of all respondents)	3.08	10.38
Per household average loss (% of livestock owned by respondent)	0.58	4.13

### 3.4 Relationship between snow leopard, wild prey and livestock density

**Figure 2:**
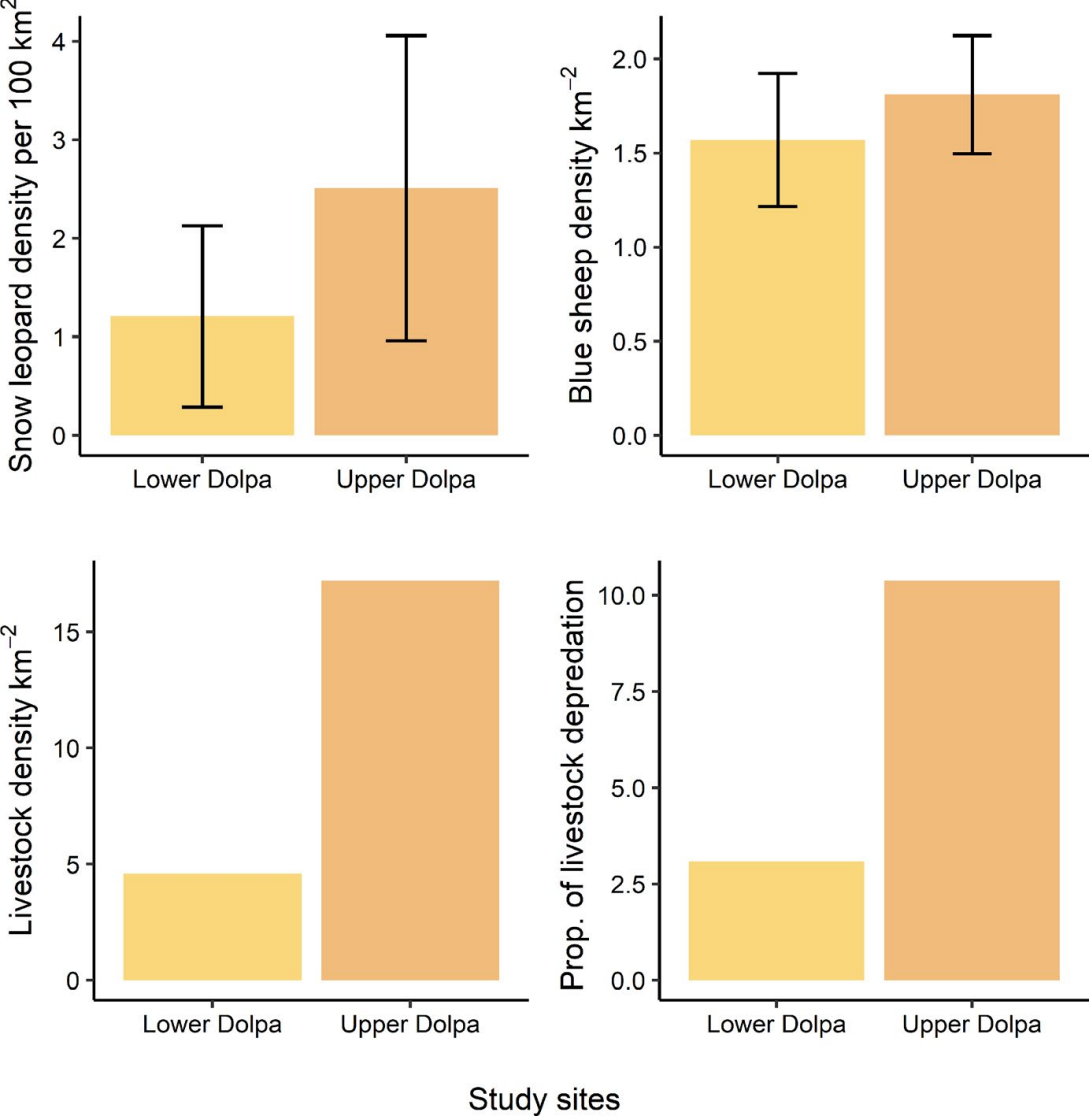
Comparison of estimated density of snow leopard based on spatial capture–recapture analysis, wild prey blue sheep density, livestock density, and proportion of livestock holding lost to snow leopard across two sites (Upper Dolpa and Lower Dolpa) in Shey Phoksundo National Park, Nepal. Proportion of livestock depredation was calculated as total livestock lost in a calendar year 2017 by all respondents divided by sum total of the livestock holding owned by all respondents

The blue sheep density was similar in both sites. The 95% confidence intervals around density estimates for the two sites overlapped. The estimated snow leopard density, livestock number, livestock density, and average livestock holding were all higher in Upper Dolpa in comparison to Lower Dolpa (Figure 2). The proportion of livestock loss per household (total livestock lost to snow leopards divided by total livestock holding owned by a respondent household) and proportion of total livestock holding lost (total livestock lost by all respondents divided by the sum total of livestock holdings of all respondents) in a year was significantly higher in Upper Dolpa (Table 4, Figure 2).

### 3.5 Factors influencing household level variation in livestock depredation

Respondents who owned higher number of livestock were likely to incur more livestock loss to snow leopard (*β* = 0.45 ± *SE* 0.03). Greater number of grazing days in pasture increased the chances of a respondent losing a greater number of livestock to snow leopards (*β* = 0.46 ± *SE* 0.04). The respondents with higher number of small bodied livestock in their livestock holding composition lost significantly higher number of livestock to snow leopards (*β* = 0.85 ± *SE* 0.06). Number of large bodied livestock in total livestock holding size did not have a statistically discernable impact on household level livestock loss to snow leopard (*β* = 0.0009 ± *SE* 0.04; Table S4).

## 4 DISCUSSION

Retaliatory killing over livestock depredation is a serious threat to survival of large mammalian carnivores. We compared whether livestock predation by snow leopards differs in relation to livestock and wild prey densities in two sites. We found higher extent of snow leopard predation on livestock both in terms of proportion of total livestock holding lost (of all sampled households) and per household loss in Upper Dolpa—the site which had higher estimated snow leopard and livestock density, and higher abundance of livestock but similar wild prey density compared to Lower Dolpa. The combined influence of higher snow leopard and livestock density appeared to have resulted in greater livestock predation in Upper Dolpa. This also suggests that an increase in livestock population and/or snow leopard population could potentially intensify predation. Previous studies have suggested that livestock predation by snow leopards may increase with increase in livestock stocking density and also with an increase in wild prey via increased abundance of snow leopards (Chetri et al., 2019; Suryawanshi et al., 2013).

We found higher snow leopard density in Upper Dolpa despite similar wild prey densities in both the sites. This is surprising because wild prey density is known to be a critical determinant of snow leopard density (Suryawanshi et al., 2017). Upper Dolpa is a trans‐Himalayan site with higher livestock population and density. Whether the Upper Dolpa (Trans‐Himalayan site) is more suitable habitat for snow leopards, or whether there were other threats or constraints for snow leopard in Lower Dolpa (the Himalayan site), needs to be further investigated.

Factors such as spatio‐temporal availability of livestock, livestock husbandry practices and habitat characteristics (e.g., relative availability of terrain ruggedness) are also important drivers of livestock predation. Previous studies on snow leopard predation on livestock show that combination of lax herding practices and availability of wild prey affects livestock predation (Bagchi & Mishra, 2006; Hussain, 2003; Jackson et al., 1996; Johansson et al., 2015; Oli et al., 1994), with straggler livestock left behind during grazing being highly preyed upon (Johansson et al., 2015). While we were unable to explicitly quantify the difference in spatio‐temporal availability of the livestock in our study sites, accounts from local herders revealed that livestock holding size and distribution of grazing pastures differed between two sites, with Upper Dolpa in particular having higher livestock density, higher average livestock holding size, more sparse human settlements, more small stocks (sheep & goats), higher number of grazing days in pastures far from human settlements than Lower Dolpa. It is thus possible that these differences in livestock availability could have also influenced the extent of depredation across the two study sites as shown in other studies (Ghoddousi et al., 2016; Khorozyan et al., 2017).

We found household who owned more livestock and a greater proportion of small bodied livestock to lose higher number of livestock to snow leopard predation. Large herd size with higher number of small bodied livestock may be easier to detect and prey upon (Chetri et al., 2019; Mijiddorj et al., 2018). We found that herders who grazed their livestock for longer durations (number of days) in pastures were likely to lose more livestock over the year to snow leopards. Number of large bodied livestock in holding size did not have significant influence on livestock predation, unlike a previous study in Central Nepal that showed households owning greater proportion of large bodied livestock to more likely incur livestock loss (Chetri et al., 2019). This could be because herders in our study site may guard large stock actively due to their higher economic value. It is also possible that higher availability of small bodied livestock may offset some level of predation pressure on large bodied stock.

Retaliatory killing of large carnivores is one of the most pressing conservation threats, and livestock depredation is the major driver of such persecution of carnivores. Minimizing retaliatory killings is thus essential for large carnivore conservation. Our findings, which indicate greater livestock predation at higher snow leopard density and livestock density, and herders with greater proportion of small bodied livestock who also graze in pastures for longer duration over the year, losing more livestock, have implications for management of conservation conflicts and retaliatory killings over livestock predation. The main implication of our study is that human snow leopard conflict management strategy needs to focus on preventive measures because increase in snow leopard population due to conservation efforts or increase in livestock population due to local and regional demands will result in increased predation on livestock. If unmanaged, this will lead to greater conflict and retaliatory killing of snow leopards. Snow leopard habitats are multi‐use landscapes, where a growing human population will continue to depend on pastoralism (Johansson et al., 2016; Mishra et al., 2009). Across snow leopard distribution range in the Himalaya and Central Asia, livestock population is expected to increase, particularly goat population, in response to the international demand for cashmere (Berger et al., 2013). On the one hand, this increase in livestock population, while being important for local economies, may have negative impact on wild prey population as it been found to depress herbivores prey population (Mishra, Van Wieren, Ketner, Heitkonig, & Prins, 2004, Suryawanshi, Bhatnagar, & Mishra, 2010). The resulting outcomes of depressed wild prey population could result in reduced density of snow leopards. On the other hand, conserving snow leopards, while being positive for conservation, may have repercussions on local economies that rely on livestock production, as higher density of snow leopards may also imply higher livestock predation, and consequently more retaliatory killing of snow leopards. Conservation initiatives aiming to recover snow leopard populations or efforts to increase livestock populations in multi‐use landscapes for enhancing local economies therefore must be accompanied by preventive measures to protect livestock predation by snow leopards (e.g., predator‐proof corrals), and offsetting economic costs of livestock predation (e.g., compensation payments, community managed livestock insurance programs).

## CONFLICTS OF INTEREST

None.

## AUTHOR CONTRIBUTION

**Gopal Khanal:** Conceptualization (equal); Data curation (lead); Formal analysis (lead); Methodology (equal); Writing‐original draft (lead); Writing‐review & editing (equal). **Charudutt Mishra:** Conceptualization (equal); Methodology (equal); Supervision (supporting); Validation (equal); Writing‐review & editing (equal). **Kulbhushansingh R. Suryawanshi:** Conceptualization (equal); Formal analysis (supporting); Methodology (equal); Supervision (lead); Validation (equal); Writing‐original draft (supporting); Writing‐review & editing (equal).

## Supporting information

Supinfo

## Data Availability

All the relevant data used in this study have been archived in Dryad https://doi.org/10.5061/dryad.dfn2z34zq
