# Supplementary material for: Relative influence of wild prey and livestock abundance on carnivore‐caused livestock predation
Source: Ecol Evol. 2020 Sep 24;10(20):11787–97. doi: 10.1002/ece3.6815 (PMC7593152; doi:10.1002/ece3.6815)
Supplement: Supplementary file 1 — Supinfo [file ECE3-10-11787-s001.docx]

**Supplementary information**

Supplementary information (S1). S1a. The final set of a priori spatially explicit capture-recapture models fitted for snow leopards capture recapture data from camera trap surveys in Lower Dolpa, November 2017- February 2018

| **D^a^** | **g0^a^** | **σ^a^** | ***K*^b^** | **AIC*_c_*^c^** | **ΔAIC*_c_*^d^** | ***w_i_*^e^** | **Deviance^f^** |
| --- | --- | --- | --- | --- | --- | --- | --- |
| Constant | Constant | constant | 3 | 560.71 | 0.00 | 0.83 | -273.36 |
| Constant | Bk | constant | 4 | 564.48 | 3.77 | 0.13 | -268.24 |
| Constant | B | constant | 4 | 566.69 | 5.98 | 0.04 | -269.34 |
| Constant | b+T | constant | 5 | 608.10 | 47.38 | 0.00 | -269.04 |
| Constant | B | b | 5 | 608.12 | 47.41 | 0.00 | -269.06 |

S1b. The final set of a priori spatially explicit capture-recapture models for snow leopards fitted for capture recapture data from camera trap surveys in Upper Dolpa, November 2017- February 2018

| **D^a^** | **g0^a^** | **σ^a^** | ***K*^b^** | **AIC*_c_*^c^** | **ΔAIC*_c_*^d^** | ***w_i_*^e^** | **Deviance^f^** |
| --- | --- | --- | --- | --- | --- | --- | --- |
| Constant | Constant | constant | 3 | 539.81 | 0.00 | 0.63 | -265.70 |
| Constant | Bk | constant | 4 | 542.14 | 2.34 | 0.19 | -264.85 |
| Constant | B | constant | 4 | 542.82 | 3.02 | 0.14 | -265.19 |
| Constant | b+T | constant | 5 | 547.23 | 7.43 | 0.01 | -264.86 |
| Constant | B | b | 5 | 547.77 | 7.97 | 0.01 | -265.13 |

^a^Density (D), detection probability at the activity center (g0), and the spatial scale over which g0 declines (σ) a function of animal × detector learned response (bk); permanent learned responses across all detectors (b); time trend (T); + = additive effect; constant = no variation.

^b^Number of model parameters.

^c^Akaike’s Information Criterion adjusted for small sample size.

^d^The difference between the top ranked model and the *i*th ranked model.

^e^Model weight. ^f^Model deviance = −2(log-likelihood

**Supplementary information (S2)**

Since snow leopard camera trap data and wild prey data were collected at a little bit different time periods, we were unsure if relating snow leopard density to wild prey density would be as scientifically rigorous as it should be. Therefore, to check if snow leopard occupancy remains constant over the two time periods (November 2017-January 2018) and (February, 2018-April, 2018), we run the analysis with standard occupancy model (Mackenzie et al. 2002). The 95% confidence interval of the occupancy estimates for two time period overlapped suggesting that occupancy status of snow leopard remained fairly constant over two time periods. The estimated occupancy for November 2017-January 2018 is Ψ =0.81 (95% CI=0.60-0.92) and for February 2018-April 2018 is Ψ =0.85 (95% CI=0.62-0.95).

S2 Table 1. Results of the basic occupancy models run with two data sets of different seasons to examine if the parameter estimate (occupancy Ψ) remained constant across two time periods.

| **Season** | **Model** | **AIC** | **deltaAIC** | **AIC wgt** | **Model Likelihood** | **no.Par.** | **Deviance** |
| --- | --- | --- | --- | --- | --- | --- | --- |
| November 2017- January 2018 | psi(.),p(.) | 441.27 | 0 | 1 | 1 | 2 | 437.27 |
| Feburary 2018- April 2018 | psi(.),p(.) | 442.84 | 0 | 1 | 1 | 2 | 438.84 |

**Supplementary information (S3)**

**Data form used to collect information on livestock depredation by snow leopard and household characteristics of the respondent.**

Surveyor Name: Date: Data form No:

1. Respondent’s name: Address: Sex: Age:
2. Household size: Male ( ) Female ( ) Education:
3. Occupation: Income sources:
4. Livestock population: Yak ( ), Yak-cattle hybrid ( ), Goat ( ), Sheep ( ), Others ( )
5. Have you lost any livestock to snow leopard over the year Jan-Dec 2017?, If yes, please report each of the livestock depredation case in detail as follows:

| Date of the case | Pasture/location of livestock lost | Age and species of livestock | Time of the predation event | Remarks |
| --- | --- | --- | --- | --- |
|  |  |  |  |  |
|  |  |  |  |  |

1. What type of husbandry practices have you practiced to prevent livestock depredation by snow leopard?
2. Herding b) use of guard dogs c) corral d) any other
3. Do you have a predator proof corral? If yes, how many?
4. How many days do you graze your livestock in pastures?

**Supplementary information (S4)**

We examined the influence three household factors in household level variation in number of livestock lost to snow leopard. These factors are: livestock holding size of the household, number of grazing days in pasture and proportion of the small bodied livestock owned by a household. Since the response variable was count data, we run Generalized Linear Models (GLMs) with a Poisson error distribution and log link function. We developed six plausible models to explain variation in household level livestock depredation (S4 Table, 1). Proportion of small bodied livestock owned by a respondent and total livestock holding size were correlated (r=0.50, p<0.05), and hence were not included in same model.

We ranked models using the Akaike information criterion adjusted for small samples (AICc; [Burnham & Anderson, 2002](#b8)). Models with ΔAIC<2 were considered to be strongly supported by the data. All analyses were carried out in *R 3.4.2* ([R Development Core Team, 2017](#b32)). Model selection results show that additive influence of number of small bodied livestock and grazing days in pasture had the lowest AICc and highest Akaike model weight suggesting this model has the highest chance of being the bed model among the candidate models examined (S4 Table 1). Number of livestock lost at household level was positively associated with number of small bodied livestock, total number livestock and grazing days in pasture whereas number of large bodied livestock did not have any significant influence on livestock depredation (fig. Sa, Sb, Sc, Sd).

S4. Table 1. Poisson regression models explaining the livestock depredation by snow leopard across sampled households over a year 2017, ranked according to the Akaike information criterion adjusted for small sample size (AICc).

| Model^1^ | *K*^2^ | AICc | ΔAICc^3^ | Loglink | *W_i_*^4^ |
| --- | --- | --- | --- | --- | --- |
| Number small bodied livestock+ Grazing days in pasture | 3 | 797.33 | 0.00 | -395.59 | 1.00 |
| Total livestock number+ Grazing days in pasture | 3 | 824.01 | 26.68 | -408.93 | 0.00 |
| Number of small bodied livestock | 2 | 841.68 | 44.35 | -418.81 | 0.00 |
| Livestock number | 2 | 875.08 | 77.75 | -435.51 | 0.00 |
| Grazing days in pasture | 2 | 972.28 | 174.95 | -484.10 | 0.00 |
| Grazing days in pasture+Number of large bodied | 3 | 974.15 | 176.82 | -484.00 | 0.00 |
| intercept only | 1 | 1063.99 | 266.66 | -530.98 | 0.00 |
| Number of large bodied | 2 | 1065.98 | 268.65 | -530.95 | 0.00 |

^1^Name of model

^2^Number of parameters.

^3^Difference between the AICc value of the best-supported model and successive models.

^4^Akaike model weight.


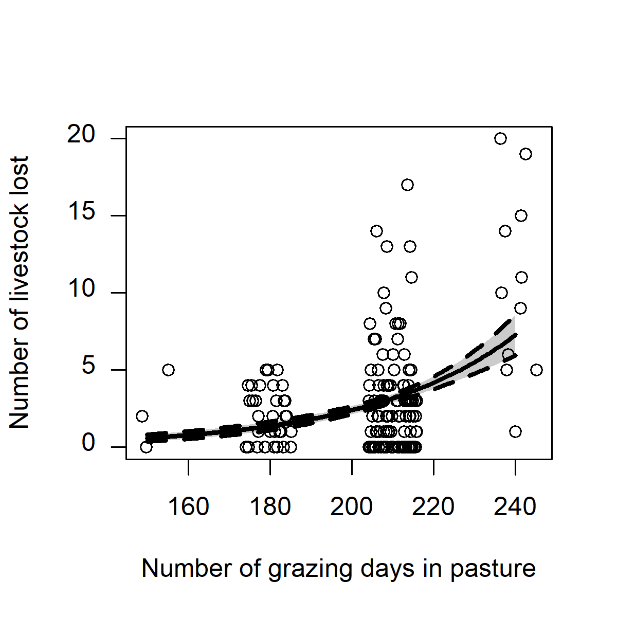

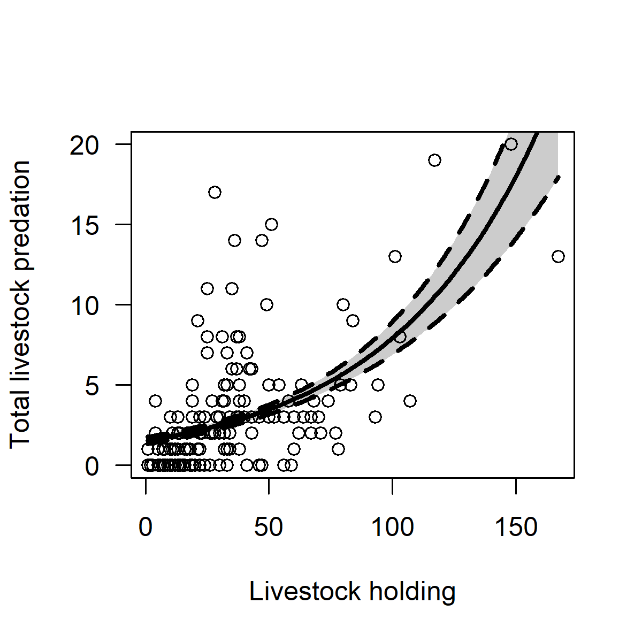


**b.**

**a.**


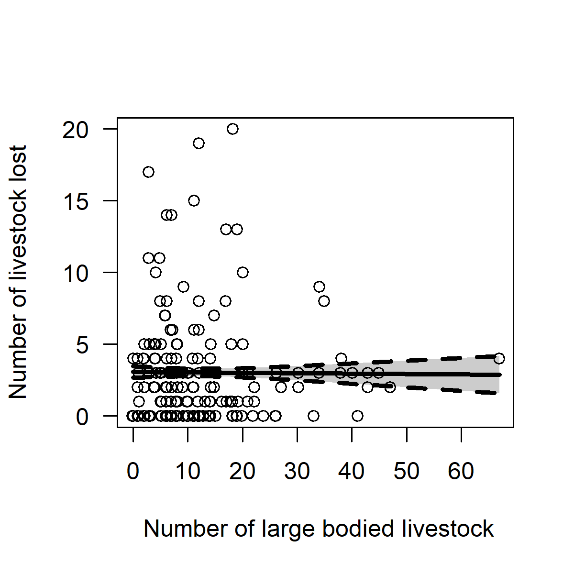


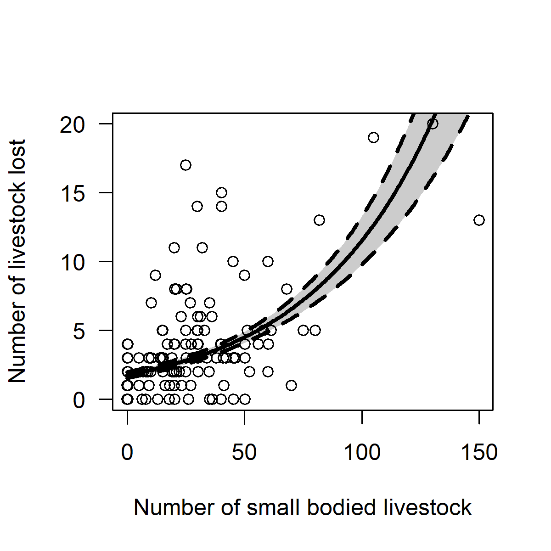


**c.**

**d.**

Fig. Sa, Sb, Sc. Predicted relationship between livestock loss per household and number of grazing days in pastures, livestock holding owned by a respondent and proportion of small bodied livestock owned by a respondent based on Generalized Linear Models (GLMs).
